# Difficulties with tunneling of the cuffed catheter: a single-centre experience

**DOI:** 10.1038/s41598-018-21338-5

**Published:** 2018-02-20

**Authors:** Tomasz Gołębiowski, Mariusz Kusztal, Krzysztof Letachowicz, Jerzy Garcarek, Tomasz Porażko, Jan Penar, Magdalena Krajewska, Wacław Weyde, Marian Klinger

**Affiliations:** 1 Department of Nephrology and Transplantation Medicine, Wroclaw Medical University, ul.Borowska 213, 50-556 Wroclaw, Poland; 2 Department of Radiology, Wroclaw Medical University, ul. Borowska 213, 50-556 Wroclaw, Poland; 3 Department of Nephrology Voivodoship Medical Center Opole, Al Witosa 26, 45-418 Opole, Poland

## Abstract

Tunneling of the cuffed catheter for hemodialysis is an important part of insertion procedure with faulty techniques being the cause of catheter dysfunctions. We retrospectively analyzed 737 double-lumen cuffed catheter procedures between 2008 and 2015 in patients aged 60 ± 15years, requiring renal replacement therapy. Complications of tunneling included kinking, bleeding and other problems. In 20 of 737 (2.7%) procedures, the catheter kinked, which was observed in 7.7% of silicone and 0.6% of polyurethane catheters. Repositioning was attempted in 4, but was successful in only 2 cases. Catheter exchange was necessary in 16 cases, but the function was adequate in 2 cases, despite radiological signs of kinking. In 6 cases (1 patient with diabetes, 2 with chest anatomy changes and medical devices, 2 with systemic sclerosis and 1 with greatly enlarged superficial jugular veins) we faced particular difficulties requiring an individual solution by tunneling; these are described in detail. The cumulative catheter patency rate were 69%, 52% and 37% at 3, 6 and 12 months, respectively. In conclusion, the most frequent complication of tunneling was kinking, usually necessitating catheter exchange. The silicon catheter kinked more often than the polyurethane one. An individual approach is sometimes needed by patients with diabetes and anatomical changes of the chest.

## Introduction

Cuffed hemodialysis catheters are used as vascular access in one third of the chronic dialysis population^1,2^ usually to bridge the vascular access until autogenous or non-autogenous access can be established. These catheters were developed more than three decades ago^3^ and are still used all over the world because of a significant reduction of infectious complications in comparison to temporary catheters^4^. The insertion technique of the long-term catheter is sometimes a challenge for the physician. An important part of this procedure is tunneling, which involves the catheter being conducted through subcutaneous tissues from the exit site usually in the infraclavicular area to the point on the neck where the guide wire was introduced into the jugular vein.

In the present study, we analyze the complications of tunneling and describe six different clinical cases in which we faced difficulties in tunneling and our practical approach. To our knowledge it is the first analysis of tunneling problems resulting from inserting a cuffed catheter.

## Material and Methods

All patients in whom double-lumen permanent catheter was implanted between January 2008 and December 2015 were recruited to our retrospective analysis. The patients signed an informed consent before catheter placement or exchange. In 655 patients (393 woman and 262 men) 737 procedures of catheter insertion 545 (74%) and exchange 192 (26%) were performed. In the group undergoing catheter exchange procedures, temporary catheters were converted to permanent ones in 125 cases and permanent to permanent in 67 cases. The exchange procedure of the temporary catheter ran either in a standard fashion, i.e. over the guide wire (66 procedures) or with by means of inserting a guide wire in another part of the vein (59 procedures). Additionally, in 14 procedures of the permanent catheter exchange the fibrin sheath disruption was performed (Table 1). The complications associated with tunneling were retrospectively reviewed from medical records and our electronic database.

**Table 1:** Procedures of catheter insertion.

Number of catheter insertion	737	%
*de novo* catheter placement	545	74
conversion of the temporary to permanent catheter (the guide wire inserted in new place)	66	9
conversion of the temporary to permanent catheter in standard fashion (over the guide wire)	59	8
conversion of the permanent to permanent catheter	53	7
conversion of the permanent to permanent catheter and balloon disruption of the fibrin sheath	14	2

### The procedure

Most procedures were carried out in the operating room in sterile conditions by 3 experienced nephrologists. Only high-risk interventions were performed in an interventional radiology suit and included the following: long-term use of catheter (over 18 months), multiple (over 2) catheter exchanges in the same place, clinical signs of central vein stenosis (extremity or face swelling, presence of the dilated collateral vein on the chest wall) and predicted need of fibrin sheath disruption. No general anesthesia was used, but at the surgeon’s discretion a sedative (diazepam 2.5–5 mg IV) was given. 1% lignocaine was used for local anesthesia at the operation site. The right internal jugular vein was the preferred access route for tunneled catheters, followed by left internal vein. The femoral and subclavian vein was used in extraordinary clinical conditions. Insertion of the cuffed catheter was performed in a standard manner. The patient was lying in a Trendelenburg position to prevent the formation of an air embolism. Only the catheters with conventional anterograde tunneling were used. The vein was punctured under ultrasound guidance (Site Rite 5 device), and the guidewire was advanced into the central vein. Then, two small, about 1 cm, sections were made, one at the point of the guide wire entry on the neck and the second 3–5 cm below the clavicula depending on the catheter size and the patient’s chest anatomy. The catheter was transferred between these 2 sections in subcutaneous tissues above the clavicula. The canal was widened by dissecting forceps in case of tunneling difficulties. Finally, the catheter was inserted through the peel-away sheath. A chest X-ray was performed just after the procedure to detect any complications.

The following catheter types have been implanted in our department: GamCath Catheter - Gambro (silicone) (No = 220), HemoStar- Bard (polyurethane) (No = 348), MAHURKAR-Covidien (carbothane) (No = 118), Palindrom- Covidien (carbothane) (No = 51). The total number of polyurethane and carbothane catheters was 517.

In patients with a temporary catheter requiring renal replacement therapy longer than 3 weeks, or in whom a renal transplant was anticipated, or arteriovenous fistula was pending maturation a guidewire conversion at the preexisting site was performed. The subcutaneous tunnel was created between the section in the subclavicular area as described above and the venotomy site, where the temporal was inserted. During the exchange, the existing catheter was free at the neck entry site, and a guidewire was passed into the superior vena cava. The temporary catheter was then removed. A sheath was placed over the guidewire, through which a new catheter was placed.

In patients with a permanent catheter, in whom guidewire exchange was necessary, the procedure was performed in the same way except for two differences. First, the old catheter was accessed on the neck and cut out. The cuff was dissected and the external part of the catheter removed. The new tunnel was created, and the guidewire was passed into the remaining catheter to the central vein. Finally, the new catheter was advanced over the peel-away sheath.

All procedures of the permanent to permanent catheter exchange combined with the fibrin sheath disruption were performed in the radiology suite under fluoroscopic control. This technique was described in detail in our previous article^5^.

The procedure of repositioning the kinked catheter was performed under radiological guidance and involved accessing the catheter on the neck and widening the subcutaneous canal in order to straighten the catheter. If the length of the catheter allowed it, the line was advanced or pulled out from the central vein in order to change the catheter localization.

Ethical approval: All procedures described in this study were performed in order to provide the best vascular access for hemodialysis patients and were in accordance with the ethical standards of the institutional research committee and with the 1964 Helsinki declaration and its later amendments or comparable ethical standards. The Medical Ethical Committee of Wroclaw Medical University has approved the study (KB 388-2016).

Informed consent for inserting the catheter was obtained from all individual patients. Additionally, the photographed participants gave written consent for publishing their photographs.

### Statistical analysis

Continuous variables were expressed as mean ± standard deviation and categorical variables as frequencies (%). The catheter survival was evaluated by the Kaplan-Meier method and by life table analysis using statistical software (STATISTICA ® 12.5, StatSoft, USA). Cumulative catheter patency was the measured outcomes in our study. According to recommended standards^6^, it was calculated as the time from catheter insertion to the time of exchange or removal for any reason including completed observations (use of AVF or graft, transfer to peritoneal dialysis, transplantation, catheter related infection, death). A censored event included patients lost from observation due to him or her being transferred to another hemodialysis unit or because of the study period coming to an end.

## Results

In general, 737 procedures of cuffed catheter insertion were performed in 655 patients, 262 (40%) male and 393 (60%) female, requiring renal replacement therapy between 2008 and 2015. There were 545 new catheters and 192 exchanges. Tunneling was associated with the following complications: catheter kinking in 20 (2.7%) procedures, hemorrhage and hematoma along catheter trajectory in 5 cases (0.6%). The hemorrhage complications were clinically non-important. No patient received a transfusion or needed an dressing change. In 4 cases of kinked catheters surgical correction-reposition was done, but this maneuver solved the problem in only 2 cases and the line was sufficiently patent. The catheter exchange was necessary in 16 patients. In spite of radiological signs of kinking, their patency was adequate in 2 catheters and no additional corrections or exchanges were made. To avoid hemolytic incidents the venous pressure was observed during the hemodialysis (HD) sessions with care that it did not exceed 250 mmHg. The cumulative patency rate were 69%, 52% and 37% at 3, 6 and 12 months, respectively (Supplementary Fig. 1). The mean time of catheter use was 354 ± 422 days. The silicone catheter kinked significantly more frequently than the polyurethane/carbothane (17 of 220 (7.7%) v. 3 of 517 (0.6%)).

We faced particular problems in tunneling in 4 cases, which is described below. In 2 cases with systemic sclerosis, although problems were initially predicted, the tunneling went surprisingly smoothly. Patients’ characteristics with tunneling problems are presented in Table 2.

**Table 2:** Demographic and clinical characteristics of 24 patients with tunneling problems. Data were expressed as means ± SD (standard deviation).

**Patients’ characteristics**	**Mean**	**±SD**
Age (years)	70.5	14.7
Body surface area (m²)	1.83	0.17
BMI (kg/m²)	27.8	0.18
Systolic BP pre-HD (mmHg)	135.1	11.5
Diastolic BP pre-HD (mmHg)	76.9	6.9
**Comorbidity scores**	**Mean**	**±SD**
Charlson Comorbidity Index (CCI) score	3.7	1.5
age-adjusted CCI (aaCCI) score	6.6	2.4
Survival 1 year (%)	82,3	7
Survival 2 year (%)	65.6	13.1
**Comorbidity**	**No**	**%**
Peripheral vascular disease	10	41
Coronary artery disease	7	29
Diabetes	6	25
Congestive heart failure	5	20
Cerebrovascular disease	2	8
Chronic pulmonary disease	2	8
**Cause of CKD**	**No**	**%**
Ischemic nephropathy	10	41
Diabetic nephropathy	6	25
Glomerulonephritis	2	8
Neoplasmatic disease	2	8
Chronic pyelonephritis	2	8
Others	2	8

### Case 1

**Figure 1:**
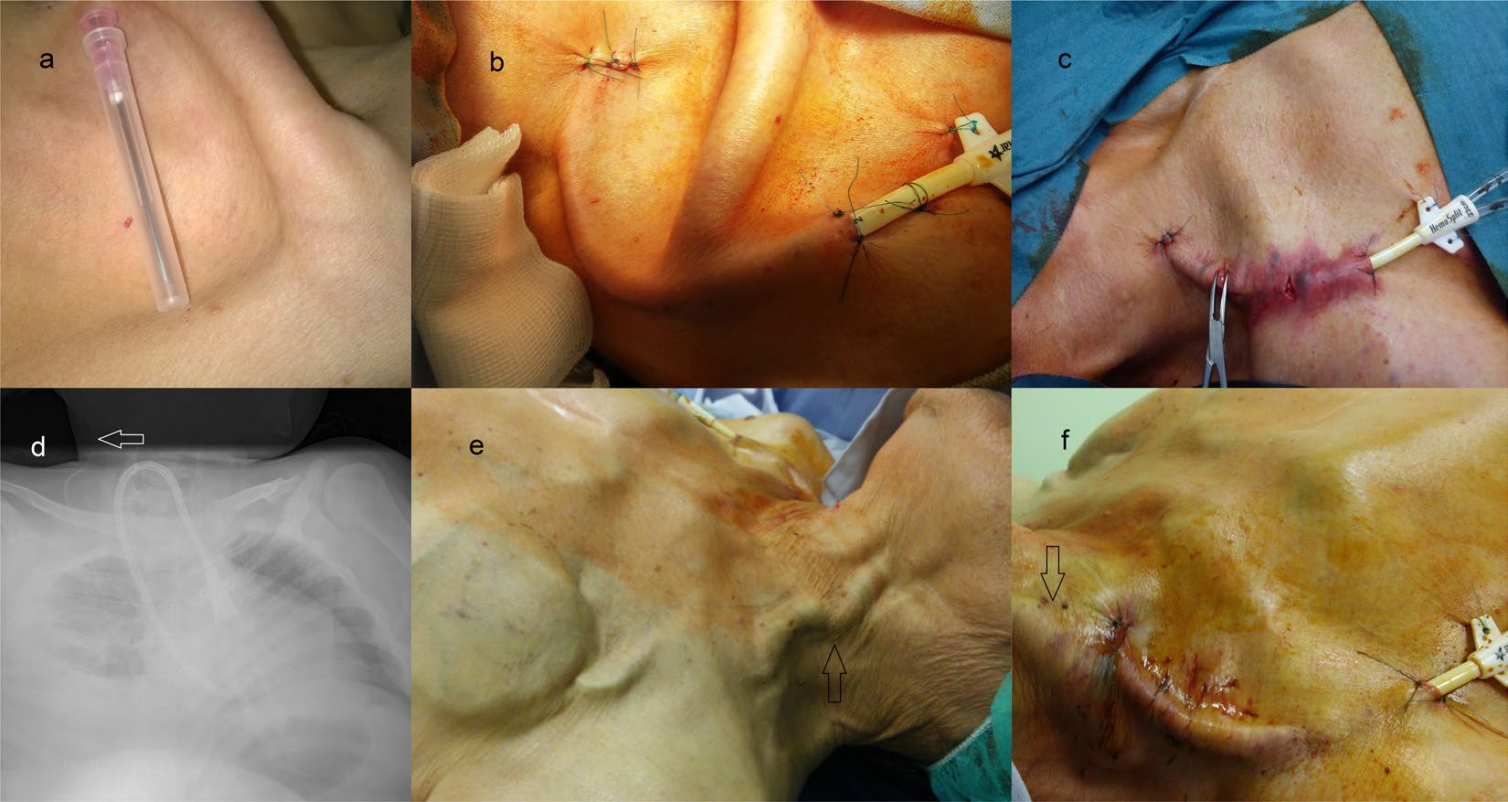
(**a**) Right supra and infraclavicular area of the chest. The skin tightly covered ribs, clavicle and scapula and (**b**) modified tunneling in hypotrophy patient. The catheter is subcutaneously conducted in the region of superior fibers of the musculus trapezius. (**c**) Tunneling in diabetic patient with stiff and inelastic skin and subcutaneous tissue. (**d**) Atypical, reverse mode of catheter tunneling.The white arrow shows a ventriculoperitoneal shunt. (**e**) Greatly enlarged superficial jugular veins (black arrows)in patient with right heart failure and (**f**) the catheter tunneling.

A 42-year-old female with chronic kidney disease stage V was admitted to our department for the creation of a vascular access and to start renal replacement therapy. The cause of kidney insufficiency was not clear. No proteinuria or hypertension was noted in her past medical history. A physical examination revealed signs of hypotrophy. Her height was 138 cm and weight 38 kg. The estimated body mass index (BMI) was 19.9 m/kg^2^. A distal forearm arteriovenous fistula (AVF) was successfully created, but after 5 days the bruit was not heard – the AVF clotted. Because of advanced kidney insufficiency we decided to insert a permanent catheter and start dialysis treatment. In typical clinical conditions, the catheter is transferred in subcutaneous tissues starting from the infraclavicular region, over the clavicle, crossing it in the middle of its length and finally reaching the point on the neck where the guide wire was introduced into the jugular vein. Advanced malnutrition with atrophy of subcutaneous tissues in our patient caused difficulty in finding a space for placing the catheter. The skin tightly covered the ribs, clavicle, and scapula, and created deep hollows in the supra- and infraclavicular area (Fig. 1a).

Keeping in mind these anatomical changes, the catheter implantation technique was modified. The jugular internal vein was punctured at the typical point (at the tip of the triangle formed by the sternal and clavicular head of the sternocleidomastoid muscle (SMM)) and the guide wire was advanced through the needle. A small incision was made 3 cm below the shoulder joint and the catheter (Hemostar, Bard, 23 cm) was tunneled atypically, more posterior than usual, in the region of the superior fibers of the musculus trapezius, reaching the exit point of the guide wire on the neck (Fig. 1b). Further stages of the catheter implementation proceeded uneventfully, in a standard manner, using a peel-away sheath. The position of the catheter tip was checked on X-ray (Supplementary Fig. 2). The patient was discharged from the hospital and has been continuing hemodialysis treatment for 2 years in satellite dialysis until she died due to pneumonia.

### Case 2

A 75-year-old patient with chronic kidney disease stage V in hemodialysis program for 7 years was admitted due to purulent infection and signs of ischemia of the left hand. The brachial–cephalic direct access was created 3 months before admission after thrombosis of the radio-cephalic wrist fistula. His medical history revealed long-term tobacco use, ischemic heart disease, coronary artery bypass grafting (CABG), chronic obstructive pulmonary disease with emphysema, obesity and insulin-dependent (type II) diabetes mellitus. Arteriography of the upper left extremity revealed steal syndrome with low blood supply to the ulnar artery and palmar arches. The radial artery was not patent. A femoral catheter was temporarily implanted. The patient received antibiotics and after overcoming the infection we decided to insert a permanent catheter as a first step and then to close the fistula to enhance blood supply to the hand. The procedure of catheter insertion to the right jugular vein proceeded in a standard manner under local anesthesia. The guide wire was inserted under ultrasound guidance. The difficulties began with tunneling. The skin and subcutaneous tissues were hard, stiff and inelastic, and the preparation was very difficult. Subcutaneous transfer of the catheter from the infraclavicular region to the neck point in one step was not possible, so we were forced to divide the whole length of tunneling into 3 segments and additionally made 2 skin incisions, one under and the second above the clavicle (Fig. 1c). Finally, we successfully transferred the catheter, passing it from one incision to another under the skin. The rest of the implementation procedure went smoothly. The wounds healed uneventfully. Finally, the fistula on the left arm was closed, which improved hand blood supply. Two months later, a wrist fistula on the right forearm was performed. The patient has been dialyzed with the catheter for 6 months, until a new fistula was adequately mature and usable for dialysis.

### Case 3

A 19-year-female patient with spina bifida and myelomeningocele after implantation of a ventriculoperitoneal shunt in childhood was admitted because of deteriorating kidney function. For hemodialysis treatment the cuffed catheter was previously implanted in the femoral vein as deformation of the chest at first look precluded the insertion into the left jugular vein (on the right side of the neck the ventriculoperitoneal shunt was located). The AVF was successfully created, but the predicted maturation time seemed too long. In the case of a progressive dysfunction of femoral catheter, another vascular access was necessary. We decided to insert a permanent catheter into the right jugular vein and transfer it subcutaneously, non-typically, creating a gentle bow in reverse mode directed medially to the sternum, to avoid injuring the shunt located laterally (Fig. 1d). The implantation procedure was uneventful, and the patient has been on hemodialysis using the catheter for 7 months, until the fistula was ready for needling. She died of urosepsis 4 months later.

### Cases 4 and 5

In the last 3 years, 2 patients with systemic sclerosis (SSc) and fast progressive kidney failure were hospitalized in our department. In both cases, inserting a long-term catheter was necessary due to the impossibility of fistula creation. Because of typical skin lesions including the upper part of the chest and neck, we predicted difficulties with catheter insertion, especially by tunneling. Surprisingly, there were no problems with transferring the catheter in the subcutaneous tissue. Both patients, however, died shortly after the initiation of dialysis treatment (in 3rd and in 4th months, respectively) due to unexpected cardiac arrest and pneumonia with decompensation of heart failure. We present these cases with the aim of underlining that skin involvement in SSc is not simultaneous with subcutaneous tissues fibrosis and there is no contradiction for a cuffed catheter in such situations.

### Case 6

In overhydrated patients or with right side heart insufficiency, a widened vein helps to puncture the vein and introduce a guidewire, but in some cases with very enlarged superficial veins, especially the external jugular vein, this may pose a problem due to the risk of injury during the creation of a subcutaneous canal (Fig. 1e). Here we present a patient with severe tricuspid valve insufficiency and chronic kidney disease stage V needing renal replacement therapy. The creation of an AV fistula was not possible due to heart failure (NYHA III) and the risk of deterioration, so we decided to place a permanent catheter. The main difficulty was to gently maneuver the dilatator so as not to damage the huge external jugular vein (Fig. 1f). The procedure was completed uneventfully, without any complications (Supplementary Fig. 3). Unfortunately, the patient suffered from cardiac arrest during HD session and died 5 months after catheter implantation.

## Discussion

Permanent catheters are invaluable for vascular access for hemodialysis. Tunneling problems during their implantation are relatively rare. In our department during a period of 12 years we have encountered such problems in only 24 cases out of 737 procedures. Kinking was the most frequent problem, occurring in 20 patients (2.7%). Similar results were presented by Wong et al., who reported kinking or pinching as the cause of early failure in 0.6% of all procedures of catheter placement^7^. In another study of Hamid et al., 10 out of 193 (4.9%) catheters kinked and were the cause of catheter malfunction^8^. Most of the authors who describe results of cuffed catheter insertion or replacement do not report tunneling related problems in detail and only concentrate on non-infection complications in the early period of dialysis, including catheter malfunction due to thrombosis or malposition of the tip^9–13^. In a recent study comparing the outcome of urgent-start of peritoneal dialysis or hemodialysis in 178 end-stage renal disease patients, the non-infection complications were observed more frequently after the insertion of a central vein catheter (CVC)(in 11 HD patients (13.4%)) than peritoneal catheter (in 3 PD patients (3.1%)). Bleeding and thrombosis were the main causes of CVC re-insertion during the first 30 days after catheter insertion. No technical problems including kinking were reported^13^.

The material used for catheter production can influence the risk of kinking, and inserting the line from soft material such as silicon in comparison to harder material such as polyurethane catheters is associated with increased risk of kinking. In our analysis, this complication was observed more frequently using a silicon catheter than polyurethane/carbothane (17 of 220 (7.7%) v. 3 of 517 (0.6%)). In 16 (80%) cases such lines had to be exchanged, and 2 were corrected by repositioning, because the blood supply was too low to perform adequate hemodialysis. In 2 (10%) catheters, kinking has no significant influence on patency. Although there is no analysis in the literature, this complication seems to be more frequent when the catheter is inserted by non-skilled physicians and when a faulty implementation technique is used. In order to reduce the risk of such complications, Song et al. recommend another technique of puncturing the internal jugular vein at a lateral access (posterior to SMM), instead of puncturing at the Sedillot’s triangle (the space between sternal and clavicular heads of SMM)^14^. This approach may lessen the number of angulations, leading to better flow rates and catheter function.

There are no recommendations regarding the correction of the pinched/kinked catheter. In most instances, the catheter had to be replaced. Some authors, however, try to correctly set the kinked catheter by open surgical mode with the help of vascular surgeons^15^. Our own experience does not advocate such an approach because of the high failure rate.

It should be emphasized that X-ray control of the catheter position just after or during the placement is the imperative in any situation. It could decrease the incidence of delayed detection of catheter malposition. The absence of such confirmation may lead to delayed catheter reposition and subsequent risk of infection during late manipulations.

We have described 6 unusual cases, because the technique had to be individually changed or we incorrectly predicted difficulties before catheter insertion.

In the diabetic patient, tunneling was a challenge because of the hardness of the skin and subcutaneous tissues. Adipose tissue fibrosis in patients with metabolic disturbances associated with obesity is well known^16^. Activation of preadipocytes, adipocyte hypertrophy, secretion of cytokines, infiltration by macrophages producing collagens, and inappropriately increased and rigid extracellular matrix are causes of either insulin resistance or change of mechanical properties in adipose tissue^16–18^. This process can promote local and systemic fibrosis involving subcutaneous tissue of the upper part of the chest, hindering soft tunneling. In such conditions a special approach was necessary relying on dividing the whole distance into sections and creating the tunnel in three steps.

In anorectic and malnourished patients (1 case) with little subcutaneous fat particularly in infra and supraclavicular fossa, it is difficult to find a place for catheter transfer over the clavicula. The second problem is the narrow chest diameter necessitating a sharper curve for tunneling increasing the risk of catheter kinking. In such cases, Wong et at al. proposed creating a two-step tunnel or using a more lateral approach to the internal jugular vein, instead of an anterolateral approach^6^. In our case, we widened the curve, placing the catheter laterally and in a region more posterior than usual of the superior fibers of the musculus trapezius (Fig. 1b). This maneuver allowed us to circumvent the clavicle region and generate smooth catheter trajectory.

Chest anatomy changes or medical devices implanted in the region of the neck and upper part of the chest sometimes restrict the available area of tunneling and necessitate a non-typical solution. A patient with meningomyelocele was an example of such a situation. In this case, the tunneling was created in reverse mode to the medial direction with the catheter placed above the sternum.

In cases of patients with scleroderma there were no problems with tunneling, although we predicted difficulties before catheter insertion. Systemic sclerosis (SSc) is a disorder involving the connective tissue, arterioles and microvessels characterized by tissue fibrosis of the skin and internal organs. In the skin, thickened dermis due to uncontrolled excessive deposition of extracellular matrix (ECM), mainly type I collagen, is a hallmark of this disease^19^. In contrast to the above-mentioned diabetic patients, the subcutaneous adipose tissues are intact in SSc, despite severe systemic skin involvement. The tunneling of the catheter did not pose a problem in our 2SSc cases.

A particular problem may arise in patients with heart failure and enormously enlarged jugular veins. Those cases demand special attention by catheter transposition in the subcutaneous space, because injury of superficial jugular veins may be a cause of severe bleeding requiring surgical intervention.

Tunneling is an important part of catheter insertion. Technical errors could be a cause of kinking and malfunctioning of the catheter, making it impossible to perform hemodialysis. In such situations exchange of the line is necessary and is associated with additional intervention. The physician should, therefore, be familiar with and skilled in this part of the procedure.

Our study had two main limitations. The first, it was a single center, non-matched and retrospective study. The single center nature could have impacted the complication rate associated with own experience with particular catheters type (silicone or polyurethane/carbothane). The second limitation of this study is the fact that only double-lumen catheter were used in our department. We do not implant a single-lumen lines, for example a twin Tesio catheter, so it is difficult to make a generalization about results for all cuffed catheters.

## Conclusion

The most frequent problem with tunneling of double-lumen cuffed catheter is kinking, often necessitating its exchange. X-ray confirmation of catheter position just after or during the catheter placement is the imperative and it should be performed in any situation. In patients with diabetes, fibrosis, which distorts subcutaneous tissue architecture, makes this step difficult. Paradoxically, patients with scleroderma and with hardened skin do not pose such difficulties. Malnutrition and patients with changed chest anatomy require an individual solution by planning the trajectory of the tunneling.

## Electronic supplementary material

Supplementary Figure 1-3

## References

[1] Rayner HC, Pisoni RL (2010). The increasing use of hemodialysis catheters: evidence from the DOPPS on its significance and ways to reverse it. Semin Dial..

[2] Aitken EL (2014). The use of tunneled central venous catheters: inevitable or system failure?. J Vasc Access..

[3] Schwab SJ, Buller GL, McCann RL, Bollinger RR, Stickel DL (1988). Prospective evaluation of a Dacron cuffed hemodialysis catheter for prolonged use. Am J Kidney Dis..

[4] Weijmer MC, Vervloet MG, Wee PM (2004). Compared to tunnelled cuffed haemodialysiscatheters, temporary untunnelled catheters are associated with more complications already within 2 weeks of use. Nephrol Dial Transplant.

[5] Watorek E (2012). Balloon angioplasty for disruption of tunneled dialysis catheter fibrin sheath. J Vasc Access..

[6] Lee T (2011). Standardized Definitions for Hemodialysis Vascular Access. Semin Dial..

[7] Wong JK (2002). Analysis of early failure of tunneled hemodialysis catheters. AJR Am J Roentgenol..

[8] Hamid (2015). Safety and Complications of Double-Lumen Tunnelled Cuffed Central Venous Dialysis Catheters: Clinical and radiological perspective from a tertiary centre in Oman. Sultan QaboosUniv Med J..

[9] Wang K, Wang P, Liang XH, Yuan FF, Liu ZS (2015). Cuffed-tunneled hemodialysis catheter survival and complications in pediatric patients: a single-center data analysis in China. Int J ClinExp Med..

[10] Wang L, Wei F, Chen H, Sun G, Yu H, Jiang A (2016). A modified de novo insertion technique for catheter replacement in elderly hemodialysis patients: a single clinic retrospective analysis. J Vasc Access..

[11] Mandolfo S (2014). Hemodialysis tunneled central venous catheters: five-year outcome analysis. J Vasc Access.

[12] Donati G (2015). PTFE grafts versus tunneled cuffed catheters for hemodialysis: which is the second choice when arteriovenous fistula is not feasible?. Artif Organs..

[13] Xu D, Liu T, Dong J (2016). Urgent-Start Peritoneal Dialysis and Hemodialysis in ESRD Patients: Complications and Outcomes. PLoS One..

[14] Song D, Yun S, Cho S (2015). Posterior triangle approach for lateral in-plane technique during hemodialysis catheter insertion via the internal jugular vein. AnnSurg Treat Res..

[15] Sampathkumar K, Ramakrishnan M, Sah AK, Sooraj Y, Mahaldhar A, Ajeshkumar R (2011). Tunneled central venous catheters: Experience from a single center. Indian J Nephrol..

[16] Buechler C, Krautbauer S, Eisinger K (2015). Adipose tissue fibrosis. World J Diabetes..

[17] Gustafson B, Gogg S, Hedjazifar S, Hammarstedt A, Smith U (2009). Inflammation and impaired adipogenesis in hypertrophic obesity in man. Am J PhysiolEndocrinolMetab..

[18] Henninger A. M., Eliasson B., Jenndahl L. E., Hammarstedt A. Adipocyte hypertrophy, inflammation and fibrosis characterize subcutaneous adipose tissue of healthy, non-obese subjects predisposed to type 2 diabetes. *PLoS One*. **9** (2014). 10.1371/journal.pone.0105262 PMC4141784 25148116

[19] Allanore Y (2015). Systemic sclerosis. Nat Rev Dis Primers.

