# Supplementary material for: Difficulties with tunneling of the cuffed catheter: a single-centre experience
Source: Sci Rep. 2018 Feb 20;8:3314. doi: 10.1038/s41598-018-21338-5 (PMC5820247; doi:10.1038/s41598-018-21338-5)

Difficulties with tunneling of the cuffed catheter: a single-centre experience

Tomasz Gołębiowski [1]*, Mariusz Kusztal [1], Krzysztof Letachowicz [1], Jerzy Garcarek [2], Tomasz Porażko [3], Jan Penar [1], Magdalena Krajewska[1], Wacław Weyde[1] and Marian Klinger [1]

1. Department of Nephrology and Transplantation Medicine, Wroclaw Medical University, ul. Borowska 213, 50-556 Wroclaw, Poland

2. Department of Radiology, Wroclaw Medical University, ul. Borowska 213, 50-556 Wroclaw, Poland

3. Department of Nephrology Voivodoship Medical Center Opole, Al Witosa 26, 45-418 Opole, Poland

*Correspondence to:

Tomasz Gołębiowski

Department of Nephrology and Transplantation Medicine,

Wroclaw Medical University,

ul. Borowska 213, 50-556 Wroclaw, Poland

E-mail: tgolebiowski@op.pl

tel. +48717332500, fax. +48717332509

Supplementary Figure 1. The Kaplan–Meier survival probability for all tunneled catheters. The cumulative patency rate were 69% (SE 2.7%), 52% (SE 3.0%) and 37% (SE 2.9%) at 3, 6 and 12 months, respectively. (SE = standard error).

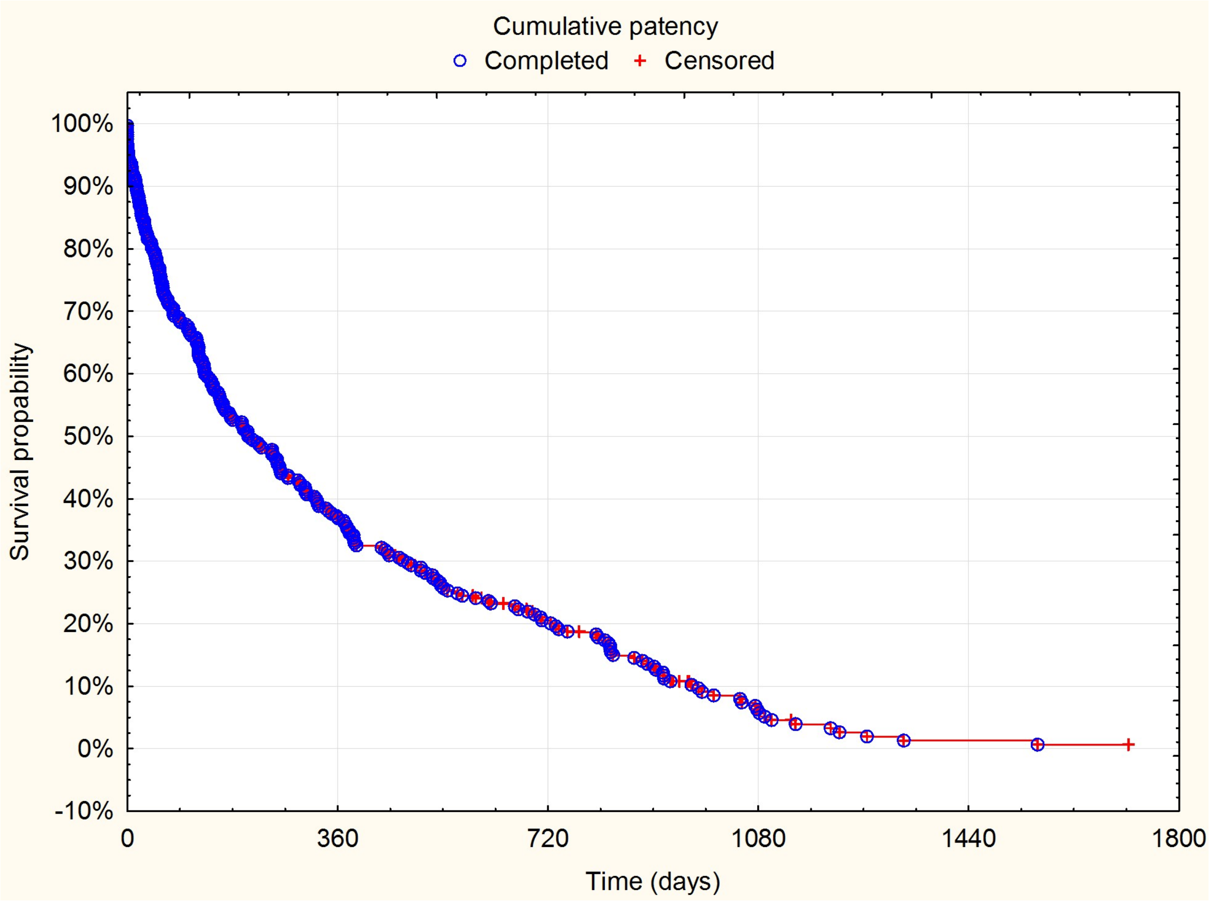

Supplementary Figure 2. Fig. 1c supp. X-ray of the chest after catheter implementation for checking the location and position of the tip.

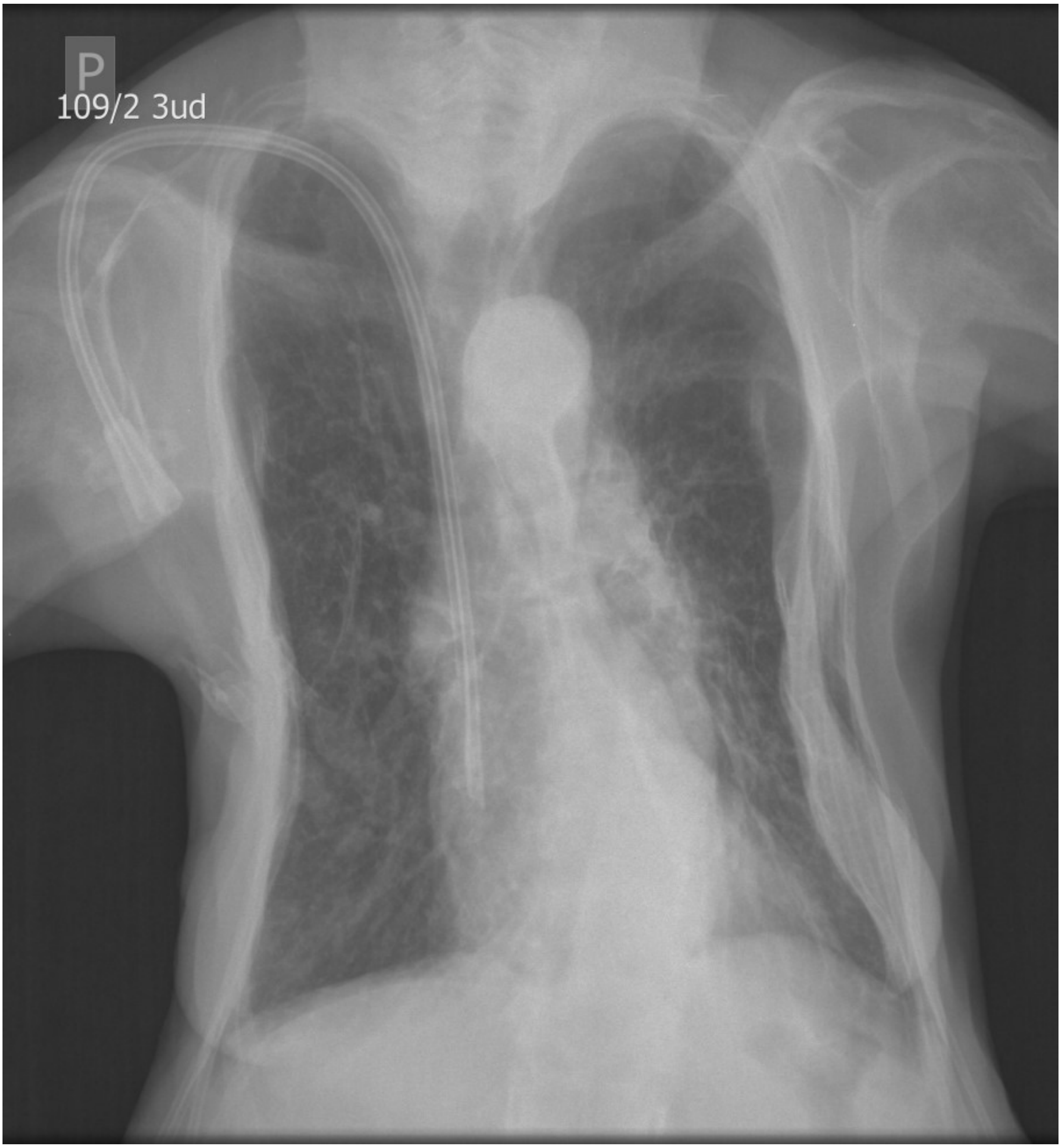

Supplementary Figure 3. X-ray of the chest in patient with right heart failure after catheter placement.

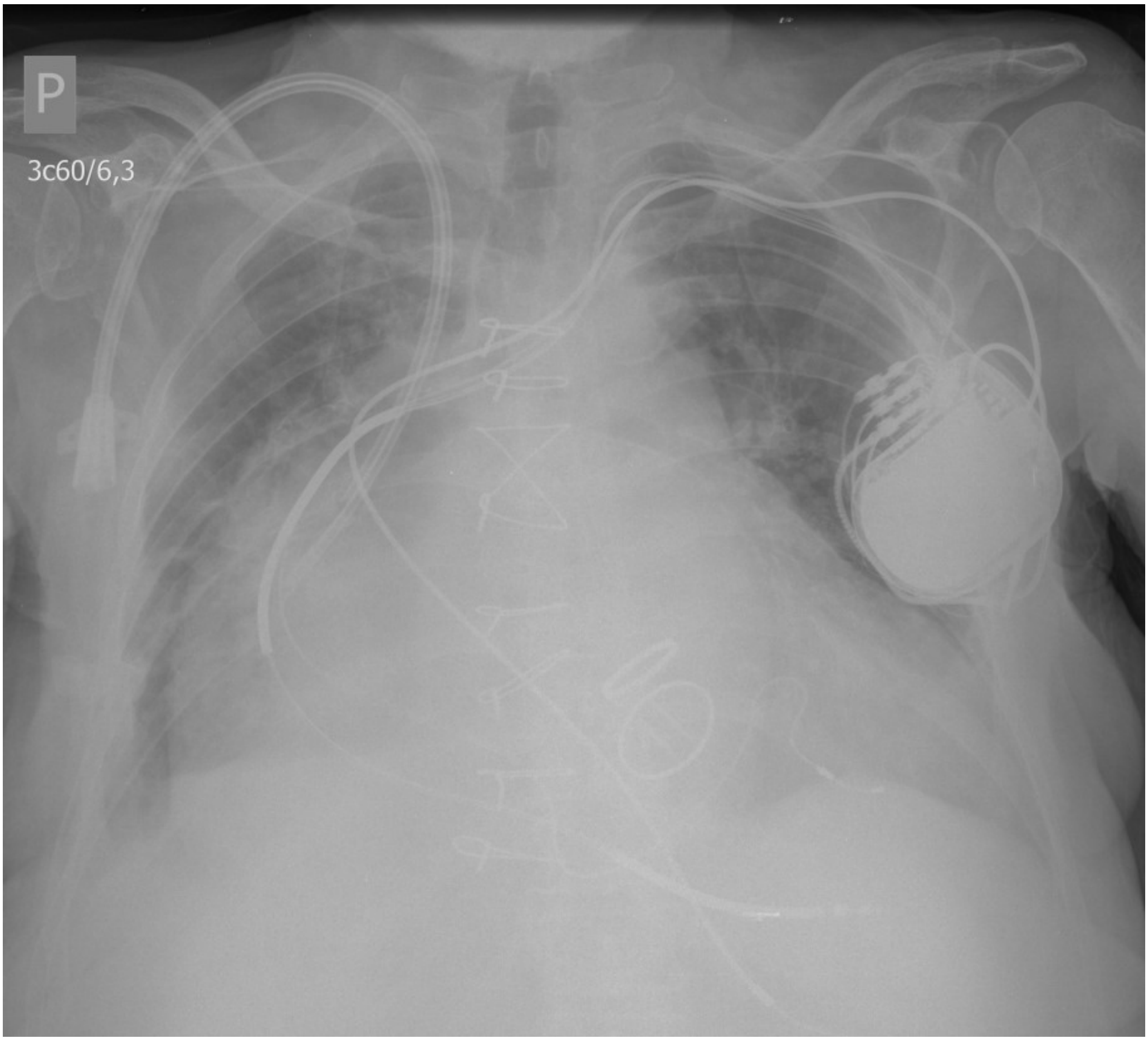

Supplement: Supplementary file 1 — Supplementary Figure 1-3 [file 41598_2018_21338_MOESM1_ESM.pdf]
